# Design and Construction of an Equibiaxial Cell Stretching System That Is Improved for Biochemical Analysis

**DOI:** 10.1371/journal.pone.0090665

**Published:** 2014-03-13

**Authors:** Chaitanya Prashant Ursekar, Soo-Kng Teo, Hiroaki Hirata, Ichiro Harada, Keng-Hwee Chiam, Yasuhiro Sawada

**Affiliations:** 1 Mechanobiology Institute of Singapore, National University of Singapore, Singapore; 2 Institute of High Performance Computing, Agency for Science, Technology and Research, Singapore; 3 Laboratory for Mechanical Medicine, Locomotive Syndrome Research Institute, Nadogaya Hospital, Kashiwa, Chiba, Japan; 4 Graduate School of Bioscience and Biotechnology, Tokyo Institute of Technology, Yokohama, Kanagawa, Japan; 5 Bioinformatics Institute, Agency for Science, Technology and Research, Singapore; 6 Department of Biological Sciences, National University of Singapore, Singapore; University of Cambridge, United Kingdom

## Abstract

We describe the design and validation of an equibiaxial stretching device in which cells are confined to regions of homogeneous strain. Using this device, we seek to overcome a significant limitation of existing equibiaxial stretching devices, in which strains are not homogeneous over the entire region of cell culture. We cast PDMS in a mold to produce a membrane with a cylindrical wall incorporated in the center, which was used to confine cell monolayers to the central membrane region subjected to homogeneous equibiaxial strain. We demonstrated that the presence of the wall to hold the culture medium did not affect strain homogeneity over the majority of the culture surface and also showed that cells adhered well onto the PDMS membranes. We used our device in cyclic strain experiments and demonstrated strain-dependent changes in extracellular signal-regulated kinase (ERK) and tyrosine phosphorylation upon cell stretching. Furthermore, we examined cell responses to very small magnitudes of strain ranging from 1% to 6% and were able to observe a graduated increase in ERK phosphorylation in response to these strains. Collectively, we were able to study cellular biochemical response with a high degree of accuracy and sensitivity to fine changes in substrate strain. Because we have designed our device along the lines of existing equibiaxial stretching technologies, we believe that our innovations can be incorporated into existing systems. This device would provide a useful addition to the set of tools applied for *in vitro* studies of cell mechanobiology.

## Introduction

Under normal physiological conditions cells experience a variety of mechanical stimuli. The consequent cell responses and adaptation to these stimuli are an important part of their normal growth, development and function. In addition to relatively passive stimuli like substrate rigidity, cells are also subjected to external mechanical forces. Three broad types of mechanical stimuli can be characterized: hydrostatic pressure, fluid shear stress, and substrate strain [1]. Cells may experience one or a combination of these stimuli depending upon their location and surrounding condition in the body. For instance, endothelial cells lining the arteries are subjected to a combination of forces, as the underlying substrate undergoes cycles of tension and relaxation and blood flow shears the cell layer. In order to study the effects of such complex forces upon cell behavior, it is desirable to isolate them and apply forces in a controlled manner *in vitro*. We focus here upon the effects of substrate strain on cell function.

To apply controlled substrate strains *in vitro*, cells are cultured on an elastic substrate which is then stretched in either a static or cyclic manner to produce a known strain field [1]. Strain applied to each elastic substrate is transmitted to the cell monolayer, and its effects are studied by either microscopy or biochemical analysis methods. The strains applied can be either uniaxial or biaxial. Uniaxial forces have been shown to produce changes in stress fiber and cell alignment [2], [3]. However, *in vitro* application of uniaxial strain may not reproduce physiological conditions. Cells *in vivo* are functionally aligned with each other, causing uniaxial strains to produce alignment-dependent effects [4]. In contrast, cells in standard culture conditions are randomly oriented. Since the cellular biochemical response to uniaxial stretching depends on the orientation of the cell relative to the direction of stretching [5], a force directed along a single axis can be experienced differently by cells without pre-orientation. Consequently, application of uniaxial strain is likely to generate mixed responses of cells in culture. Moreover, in systems applying uniaxial strain, it is difficult to avoid substrate contraction perpendicular to the stretching axis. As such, *in vitro* uniaxial stretching cannot subject cells to strains with high uniformity, which is critical for quantitative biochemical analysis of downstream signaling.

When such strain anisotropy and heterogeneity is to be avoided, systems applying equibiaxial strains are preferred. Hung & Williams [6] and Schaffer *et al.* [7] demonstrated the use of an indenter to stretch a circularly clamped elastomeric membrane to produce equibiaxial strains in the membrane. Since then, many reports have described devices that combine this method of strain application with additional features including multiple sample wells, confocal imaging, and ease of use [8]–[14]. A system that uses a vacuum to stretch the membrane is commercially available as well (FlexCell International Corp., Hillsborough, NC). These efforts have permitted significant analysis into the effects of equibiaxial mechanical stretching upon cell behavior.

**Figure 1:**
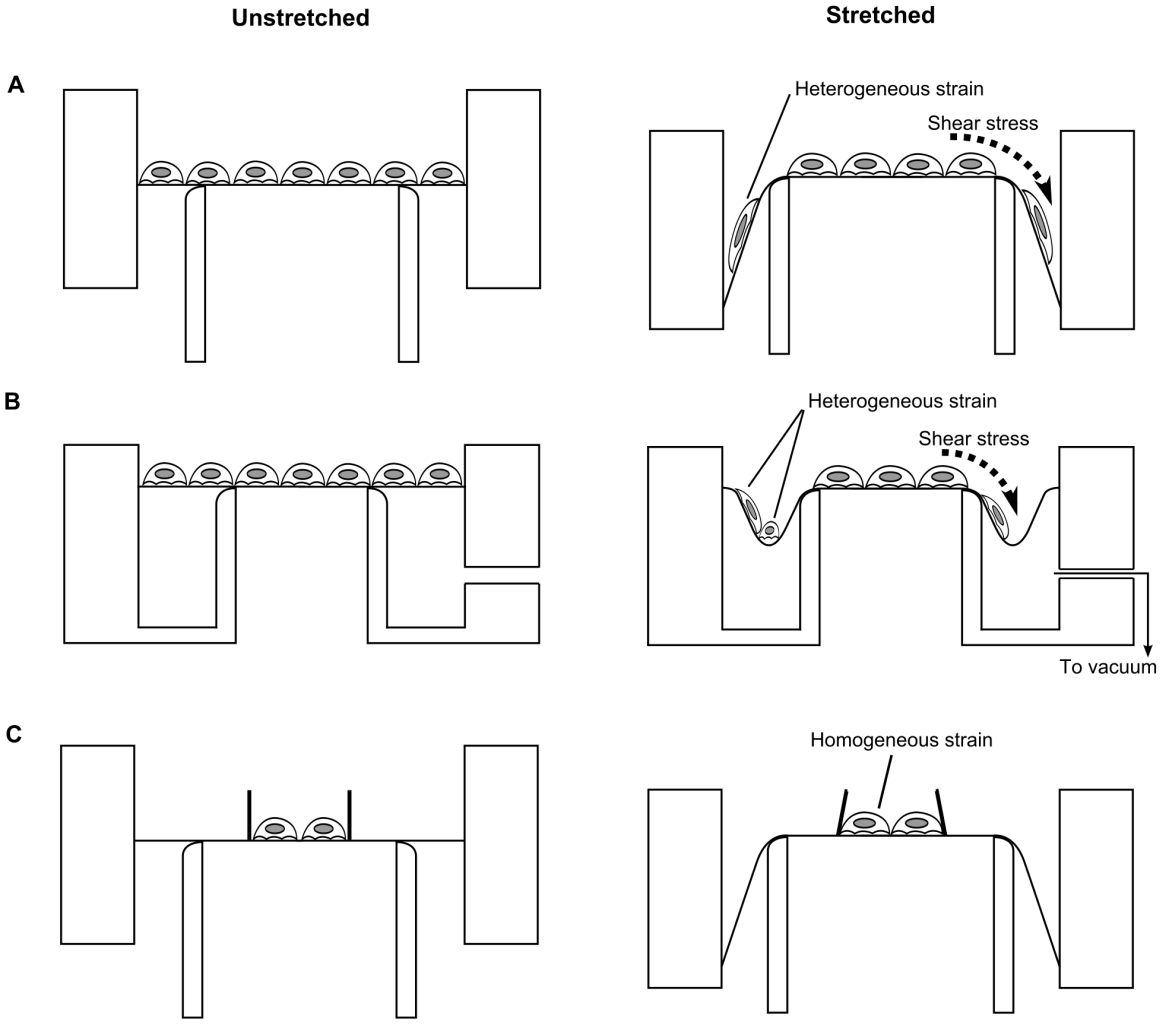
Comparison of strains imposed upon cells in different designs of equibiaxial stretching devices. (A) and (B) illustrate two representative equibiaxial cell stretching systems currently available, while (C) depicts the design that we have developed. The left and right columns correspond to the states before and after stretching, respectively. (A) When the circular clamp holding the membrane is moved downwards along the concentrically placed indenter, homogeneous strains are generated on the membrane inside the indenter. However the periphery of the membrane is stretched with different magnitudes. (B) Strain heterogeneity is also seen in the devices that adopt a vacuum to stretch the membrane. The vacuum creates a low-lying well outside the indenter, which can impart compressive forces to cells [32]. (C) When cells are confined to the central region of the membrane lying within the boundary of the indenter, they are expected to be subject to homogeneous strains.

The key advantage of such equibiaxial strain systems over uniaxial systems lies in their increased strain homogeneity. This is true for the central region of the membrane within the indenter’s circular profile. However, the outer region of the membrane lying between the indenter and the clamp does not sustain a strain field similar to that in the central region [15]. This implies that cells cultured in this region are exposed to different strains as compared to those in the central region (Figure 1A, 1B).

Such heterogeneity may be less problematic for microscopic analysis, as it is possible to exclusively image the central region of the membrane, where strains are homogeneous. However, in biochemical analysis techniques such as immunoblotting, the entire sample is collected, and the analytical results represent the average response of the cell population. It is therefore particularly important and advantageous to be able to apply uniform strain to the cell population, for quantitative accuracy and sensitivity of biochemical studies. The designs used in previously reported equibiaxial stretching devices (illustrated in Figure 1A, 1B) do not appear to subject cell populations to a sufficiently uniform strain.

The limitation described above can be resolved if the culture surface is confined to the center of the membrane, thereby only exposing cells to the homogeneous strains present in this region (Figure 1C). This idea has been implemented once before, in a system which applied in-plane stretching on a cruciform membrane to generate homogeneous strains in the central region [16]. In this paper, we improve upon these prior ideas and describe a method to confine cell culture to the center of elastomeric membranes stretched equibiaxially using an indenter.

We utilized an aluminum mold for casting poly(dimethylsiloxane) (PDMS) membranes with a cylindrical wall incorporated into the center of the membrane that essentially created a culture well. The well limited cell culture to the central region of the membrane. The custom-made membrane was incorporated into a device which is designed along the lines of previously described indenter-based stretching devices [9], [10], [12], and permitted cyclic stretching via a stepper motor and linear slide mechanism.

We confirmed that incorporation of a culture well did not interfere with the generation of uniform equibiaxial strains upon membrane stretching. The membranes could be coated with extracellular matrix proteins, and mouse embryonic fibroblasts (MEFs), as well as human embryonic kidney 293 (HEK293) cells, could be cultured on the substrates. When MEFs were subjected to varying magnitudes of cyclic stretching there was a graduated increase in ERK phosphorylation, which has been reported to be mechano-sensitive [17]–[19]. Notably, we observed stretching effects even at small magnitudes of strain (1% –6%). We also found strain-dependent changes in tyrosine phosphorylation with the sensitivity to strain magnitude varying among individual tyrosine-phosphorylated proteins.

Our system was designed to improve upon limitations in existing systems, while retaining their advantages. Using this device, we were able to perform an accurate and sensitive biochemical analysis of the effects of stretching on cell functions.

## Materials and Methods

### Finite Element Simulations

**Figure 2:**
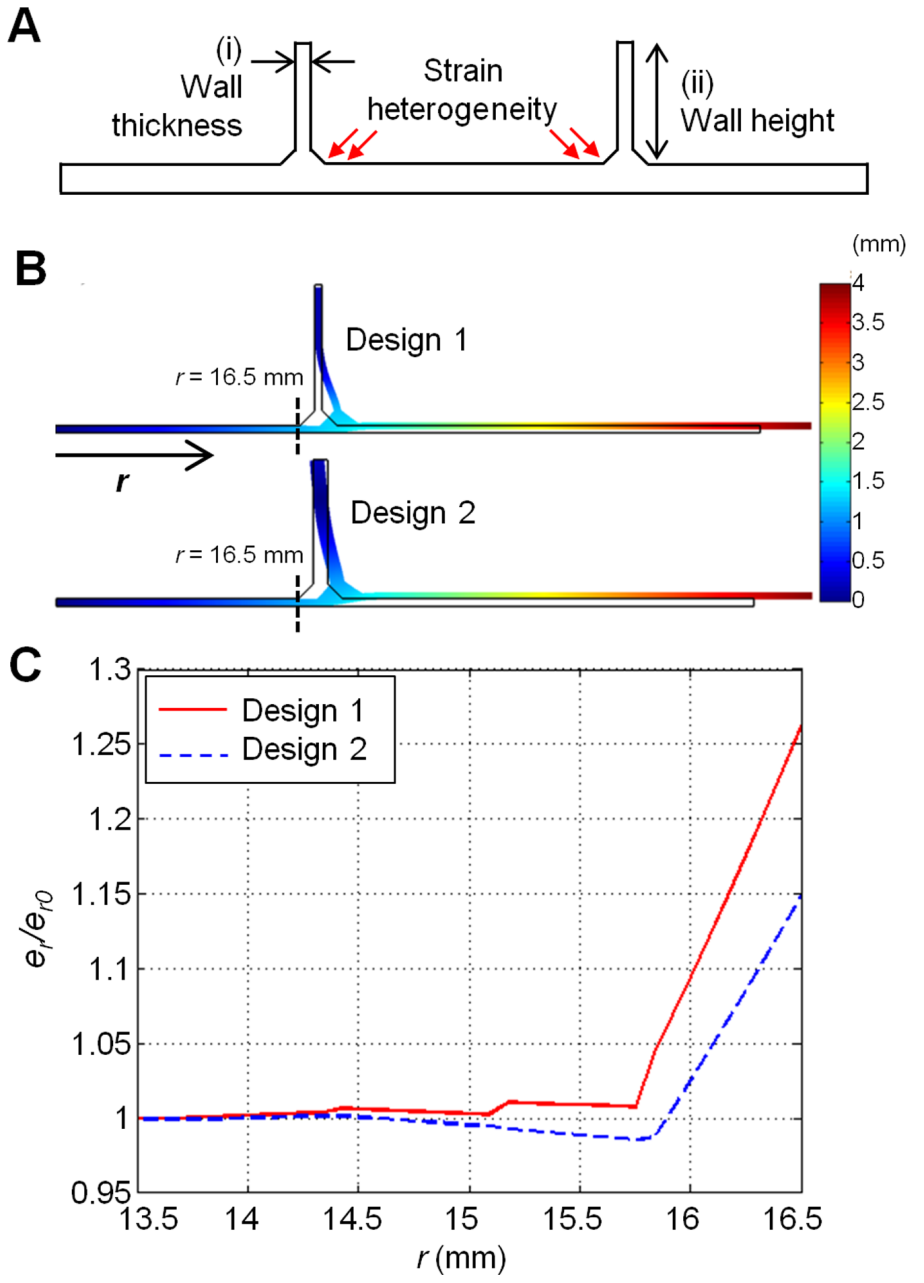
Finite element simulations to select membrane dimensions. (A) Cross sectional sketch of proposed membrane with wall (not to scale). Red arrows indicate the region near the junction of the wall and the membrane, where strain heterogeneity can arise. To reduce this heterogeneity, values for the wall thickness (i) and the wall height (ii) were selected by varying them in finite element simulations. (B) Deformation of the PDMS membrane for Design 1 (top) and Design 2 (bottom) computed by asymmetrical finite element modeling. Colors indicate magnitude of total displacement. Wall thicknesses for Design 1 and 2 are 0.5 mm and 1 mm, respectively. For both Designs 1 and 2, the thickness of the base membrane is 0.5 mm, and the wall height is 10 mm. (C) Normalized radial strain (*e_r_*/*e_r0_*) profiles for both designs near the wall-membrane junction. Design 1: *e_r0_* = 8.24%, Design 2: *e_r0_* = 7.76%. Note the smaller variation near the wall interface in Design 2 as compared to that in Design 1.

The change in membrane geometry caused by the incorporation of the wall is a potential source of strain heterogeneity. In particular, it might arise at the junction of the culture surface and the wall (see the region indicated by red arrows in Figure 2A).

Key membrane dimensions, namely: (i) wall thickness, and (ii) wall height, (Figure 2A) might influence the extent of this heterogeneity. Before constructing the mold to cast PDMS chambers, we tested a set of possible values for the wall thickness and wall height using computational analysis. We tested wall thicknesses of 0.5 mm and 1.0 mm, because thinner walls might rupture easily, while thicker walls might cause a great increase in force magnitude required to stretch the substrate. Concerning the wall height, we tested 5 mm and 10 mm, which would allow us to retain a minimum volume of medium in the chamber while avoiding excessive use of PDMS in chamber casting. The values of wall thickness and height specified above were examined in axisymmetric finite element simulations, to compute the radial (*e_r_*) strains along the culture surface of the membrane during biaxial stretching (Figure 2B). The wall thickness and wall height that gave minimal heterogeneity in *e_r_* were determined as the dimensions of PDMS chambers, and the mold was designed accordingly.

### Mold

**Figure 3:**
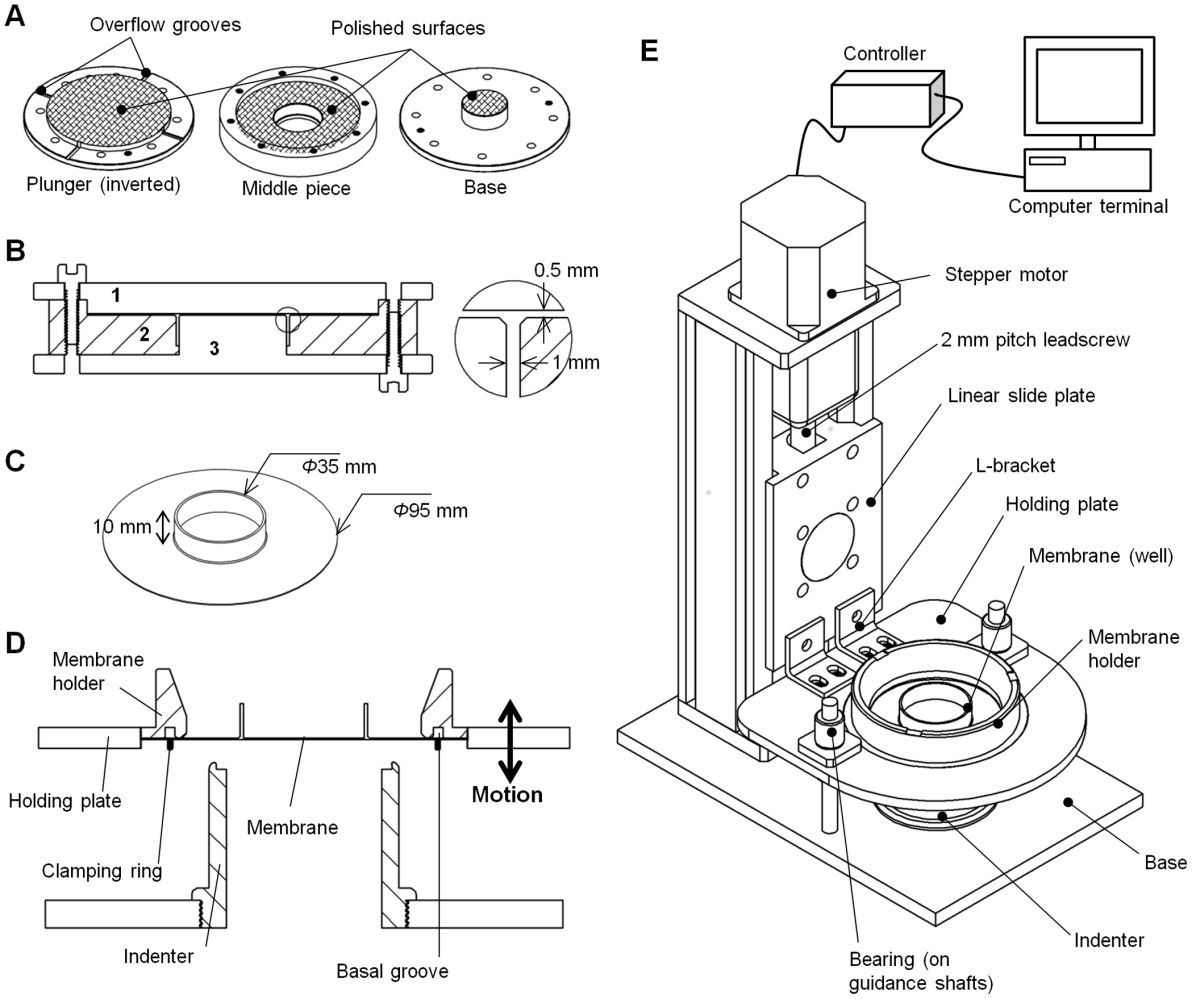
Fabrication of PDMS membranes and design of cell stretching device. (A) An aluminum mold consisting of a plunger, a middle piece and a base, was used to cast PDMS. Mold surfaces that form the base of the membrane (indicated by cross-hatching) are polished to a mirror-like finish to produce a clear surface for imaging. (B) Cross-section of an assembled mold. The base (3) and middle (2) components are assembled to form a cavity into which PDMS elastomer is poured. The top plunger (1) compresses the elastomer into the desired shape. The blowout details how mold assembly leads to the formation of a 1 mm thick wall and a 0.5 mm thick base. (C) The cured PDMS membrane has an outer diameter of 95 mm and a central well of inner diameter 35 mm. The depth of the well is 10 mm. (D) Cross-section of the membrane holder and indenter assembly. The membrane is affixed to its holder using a clamping ring made of aluminum which holds it in a circular groove at the base. Vertical motion of the holding plate leads to stretching of the membrane over the concentrically placed indenter beneath. (E) Schematic of the cell stretching device. The membrane is affixed in a membrane holder which is engaged with a holding plate. A linear slide operated by a stepper motor controls movement of the plate along a vertical axis. Bearings moving along guidance shafts constrain movement to this axis. Downward displacement of the holding plate causes membrane stretching upon a hollow indenter. The indenter, shafts, and linear slide are mounted upon an aluminum base. The motion of the holding plate is controlled by a computer.

PDMS membranes were cast using a compression molding method [20]. A three-part aluminum mold was utilized for forming the elastomer into the desired shape (Figure 3A). The two lower pieces were assembled together to form a mold cavity, into which the elastomer could be poured. The top piece served as a plunger to shape the elastomer prior to curing (Figure 3B). Mold surfaces that formed the base of the membrane were polished to a mirror-like finish (Figure 3A). This ensured that the cured PDMS had a clear surface for cell culture imaging.

### Elastomer Casting

Sylgard-184 (Dow Corning Toray, Midland, MI), a commonly available PDMS formulation, was used to create the membranes. Following the manufacturer’s protocol, base and curing agents were mixed in a 10∶1 ratio by mass. The mixture was then degassed, poured into the mold cavity, and subjected to further degassing for 45 minutes. After confirming that the elastomer had filled the cavity and completely covered the exposed surface of the mold, it was gently compressed into the desired shape using the plunger (Figure 3B). Overflow grooves on the edge of the plunger (Figure 3A) allowed any excess elastomer to be squeezed out. The entire setup was placed in an oven (80°C, 14 h) and the elastomer was cured. The mold was then disassembled and the cured membrane peeled off. Any flash arising either from the overflow grooves or due to elastomer leakage was cut off. When not in use, membranes were stored immersed in distilled water to minimize particle adsorption to the surface. The final cured solid takes the shape of a circular base of diameter 95 mm and thickness 500 µm, with a 1 mm thick and 10 mm tall cylindrical wall enclosing a central region of inner diameter 35 mm (Figure 3B, 3C).

### Stretching Device

All device parts are made of aluminum as it is resistant to rust, light weight, and affordable. The device is illustrated in Figure 3D and 3E. Membranes are clamped in a membrane holder, which is designed as a hollow cylinder with a flanged base (Figure 3D, 3E). The base is grooved, and the membrane is secured herein using a thin aluminum ring (Figure 3D). The basal flange is threaded on the outside, allowing the membrane holder to engage with a holding plate possessing a mating thread (Figure 3E) [13], [21]. The holder is screwed in until the top of its flange is level with the top surface of the holding plate. The plate is fitted with ball bearings which move along guidance shafts mounted on a base. The base holds an indenter, which is in the form of a hollow cylinder with a curved top profile (Figure 3D). Downward motion of the holding plate causes membrane stretching upon the indenter. Such downward motion is actuated by a linear slide (BiSlide, Velmex Inc., Bloomfield, NY) which connects to the holding plate by a pair of L-brackets (Figure 3E). The slide is mounted on the same base as the guidance pins and the indenter. It uses a stainless steel leadscrew to convert rotary motion to linear motion. The leadscrew is turned by a stepper motor (Vexta PK266-03, Oriental Motor USA Corp., Torrance, CA) fitted to the top of the system (Figure 3E). The motor is controlled by means of a controller (VXM-1-1, Velmex Inc.) attached to an input computer terminal.

The entire system occupies a rectangular footprint approximately 250 mm × 150 mm. The small footprint allows the device to be placed inside a cell culture incubator.

### Strain Calculation and Calibration

**Figure 4:**
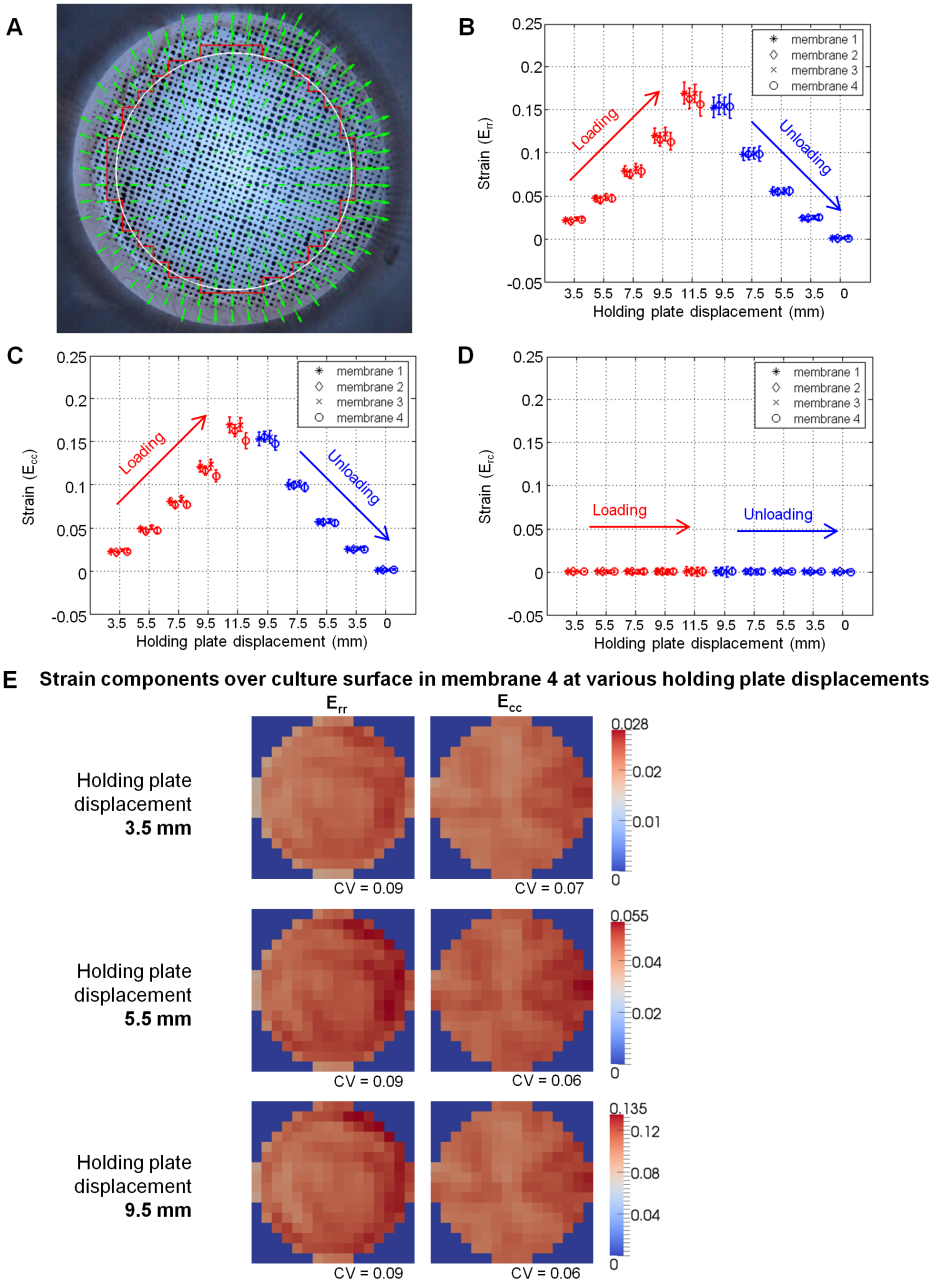
Variation of strain on the membrane surface against downward displacement. (A) A snapshot of the regular grid of displacement vectors computed using the particle image velocimetry (PIV) algorithm. This set of displacement vectors tracks the motion of the marker positions as the holding plate is displaced downwards by 3.5 mm. The white circle and the red polygonal line denote the boundary of the culture surface and the boundary for strain computation presented in (E), respectively. (B – D) Mean and standard deviation of the radial (E_rr_), circumferential (E_cc_), and shear (E_rc_) components of the strain field over the entire culture surface, respectively, plotted against corresponding downward holding plate displacement during one loading-unloading cycle. At each holding plate displacement, data points from the four membranes are horizontally staggered to allow results from individual membranes to be seen. The data points in red indicate the loading phase, during which the holding plate is being displaced downward from its initial position (0 mm) to a maximum of 11.5 mm; the data points in blue indicate the unloading phase, during which the holding plate is displaced back to its initial position. The shear strain is consistently zero regardless of the displacement of the holding plate. The curves for the radial and circumferential strains show strong overlap. Some hysteresis is seen when the membrane is unloaded; however, upon returning to the zero position, E_rr_ and E_cc_ are both consistently zero. (E) Color intensity maps of the radial and circumferential components of strain over the culture surface of membrane 4 in (B), (C), and (D) during loading phase and displaced downward by 3.5 mm (top panel), 5.5 mm (middle panel), and 9.5 mm (bottom panel). The coefficient of variation (CV), defined as the ratio of the standard deviation to the mean, is given for each intensity map.

Membrane surface strain was calculated to verify strain homogeneity. Four membranes were affixed to holders as previously described. The surface of each membrane was dotted using spray paint and a stencil to produce strain markers over the area of interest (Figure 4A). A light source and a diffuser were placed above the membrane to uniformly illuminate its surface, and a CCD camera (XZ-1, Olympus) was mounted below the system for image acquisition.

To prevent torsional strains in the membrane when the holder was screwed into the device, the initial position of the holding plate was maintained such that, when the holder was fully engaged with the plate, the lower surface of the membrane lay 2 mm above the top of the indenter. The holding plate was brought to a zero position where the lower surface of the membrane just contacted the top of the indenter, and a marker image was acquired. From here, the plate was translated downward stepwise to a peak displacement of 11.5 mm and returned to its original position along the same path. At each step on the downward and upward path, images of the surface markers were acquired.

The marker positions were tracked using a particle image velocimetry (PIV) algorithm which we implemented in MATLAB (MathWorks, Natick, USA). The principle behind PIV is pattern matching, and we used the algorithm to determine the displacement field of the markers from one image frame to the next. First, the source image was sub-divided into smaller windows, and the marker patterns within each window were correlated to all windows in the target image. The displacement field was then defined by the vector moving the window in the source image to the window in the target image with the highest correlation. This resulted in a regular grid of displacement vectors (Figure 4A).

From this displacement field, the deformation gradient tensor F_ij_, at each grid point was computed as δ_ij_+∂u_i_/∂x_j_ where δ_ij_ is the Kronecker delta second-order tensor and ∂u_i_/∂x_j_ is the gradient of the displacement field. Following this, the Lagrangian strain tensor E_ij_, was computed as 0.5*(F_ji_*F_ij_ – δ_ij_). At each step along the downward and upward path, three strain components were calculated at every point in the grid: radial strain (E_rr_), circumferential strain (E_cc_) and shear strain (E_rc_). Then, the mean and standard deviation of E_rr_, E_cc_, and E_rc_ were calculated and plotted against the corresponding membrane displacement (Figure 4B–4D).

### Membrane Preparation

Prior to use in cell culture, the membranes were immersed in 0.1 M potassium hydroxide solution and subjected to ultrasonic pulses for 30 minutes in a bath sonicator. They were then rinsed thrice with water and the sonication treatment repeated in 100% methanol. Cleaned membranes were allowed to dry in a Biosafety Level 2 (BSLII) hood until the methanol completely evaporated. The wells were rinsed thrice with sterile phosphate buffered saline (PBS) and the membranes subjected to ultraviolet irradiation for 10 minutes to sterilize the culture surface. Following this, membranes were treated with oxygen plasma for 1 minute. 1.5 ml of 25 µg/ml type I bovine collagen solution (Koken, Tokyo, Japan) was added to the plasma-activated well and incubated in a sterile, humidified, cell culture incubator (37°C, 5% CO_2_) for a minimum of 2 hours prior to cell seeding. The collagen solution was aspirated and the wells rinsed thrice with sterile PBS.

Prior to cell plating, the membranes were affixed to their respective holders. Grease (Braycote 804, Castrol Industrial North America Inc., Naperville, IL) was applied to the lower surfaces of the membranes to minimize frictional effects during membrane stretching [13], [21]. The holders were placed upon 91 mm culture plates to maintain them in a horizontal position.

After culture experiments, the membranes were washed by sonicating them in a 1% solution of detergent (Extran MA02, Merck KGaA, Darmstadt, Germany). Membranes were rinsed at least five times with water and stored in distilled water until further use.

### Membrane Biocompatibility

**Figure 5:**
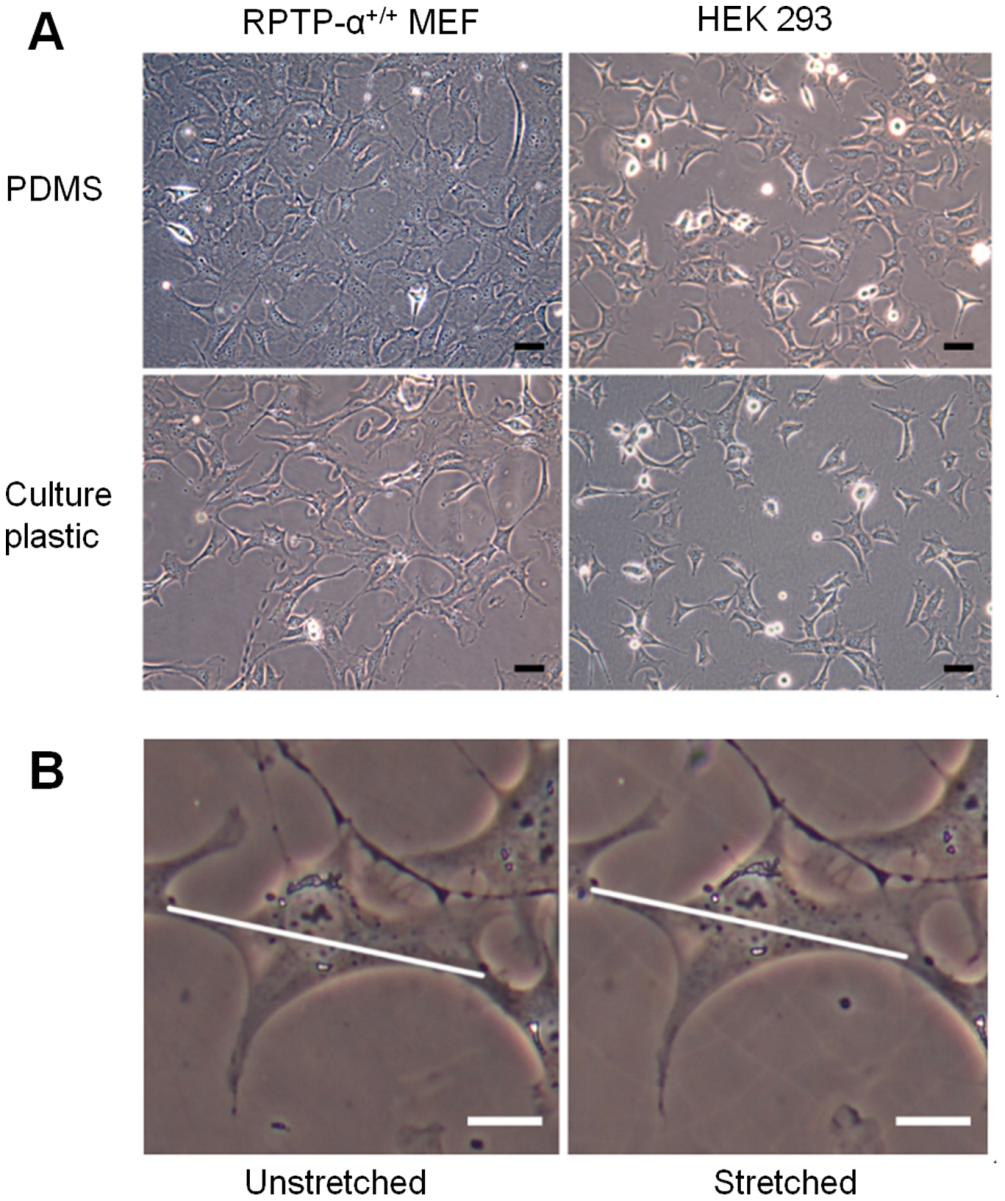
Cell culture on collagen-coated PDMS membranes. (A) Phase-contrast images (20 X) of RPTP-α^+/+^ MEFs (left) and HEK 293A cells (right), respectively. The upper row shows images of cells cultured on the PDMS membrane, while the lower row depicts cells on standard tissue-culture plastics (polystyrene). Scale bars: 50 µm. (B) Images of an RPTP-α^+/+^ MEF cell before (left) and after (right) application of 10% equibiaxial strain. The diagonal lines indicate the cell length before stretching the membrane. Note the increase of the cell dimension upon membrane stretching. Scale bars: 20 µm. The images were acquired using a manually operated device (Figure S3).

Receptor protein-tyrosine phosphatase-alpha expressing (RPTP-α^+/+^) MEFs [22] and HEK293 cells were used to confirm the biocompatibility of the PDMS membranes. Cells were maintained on standard tissue culture plastics in Dulbecco’s Modified Eagle Medium (DMEM) (Nissui, Tokyo, Japan) supplemented with 10% fetal bovine serum (GIBCO, Grand Island, NY) and 1% penicillin/streptomycin (GIBCO) were trypsinized and resuspended prior to being plated to collagen-coated membranes with density 30,000 cells/cm^2^. Phase contrast images of cells cultured for 24 hours on the membranes were acquired using an Olympus CKX41 microscope equipped with a CCD camera (E-330, Olympus) (Figure 5A).

### Cyclic Stretching Experiments

Cells were cultured to form a monolayer prior to the application of cyclic strain. The relevant strains were applied continuously for 30 minutes at a frequency of 0.5 Hz. Two types of controls were used. In the first case, a membrane holder was fitted into the holding plate and left to stand for 30 minutes prior to sample collection (Figure S1A). In the second case, the indenter was removed prior to fitting the holder. Then, the linear slide was operated with the same program as that used to generate strain of 6%, thus subjecting the cells to vertical motion without any strain (Figure S1B). For SDS-PAGE followed by immunoblot analysis, the cells were solubilized with a SDS sample buffer (62.5 mM Tris-HCl pH 6.8, 2% SDS, 5% sucrose, 0.002% bromophenol blue, 5% β-mercaptoethanol). Stretching experiments were conducted at least three times for quantification and statistical analysis.

### Immunoblotting Analysis

**Figure 6:**
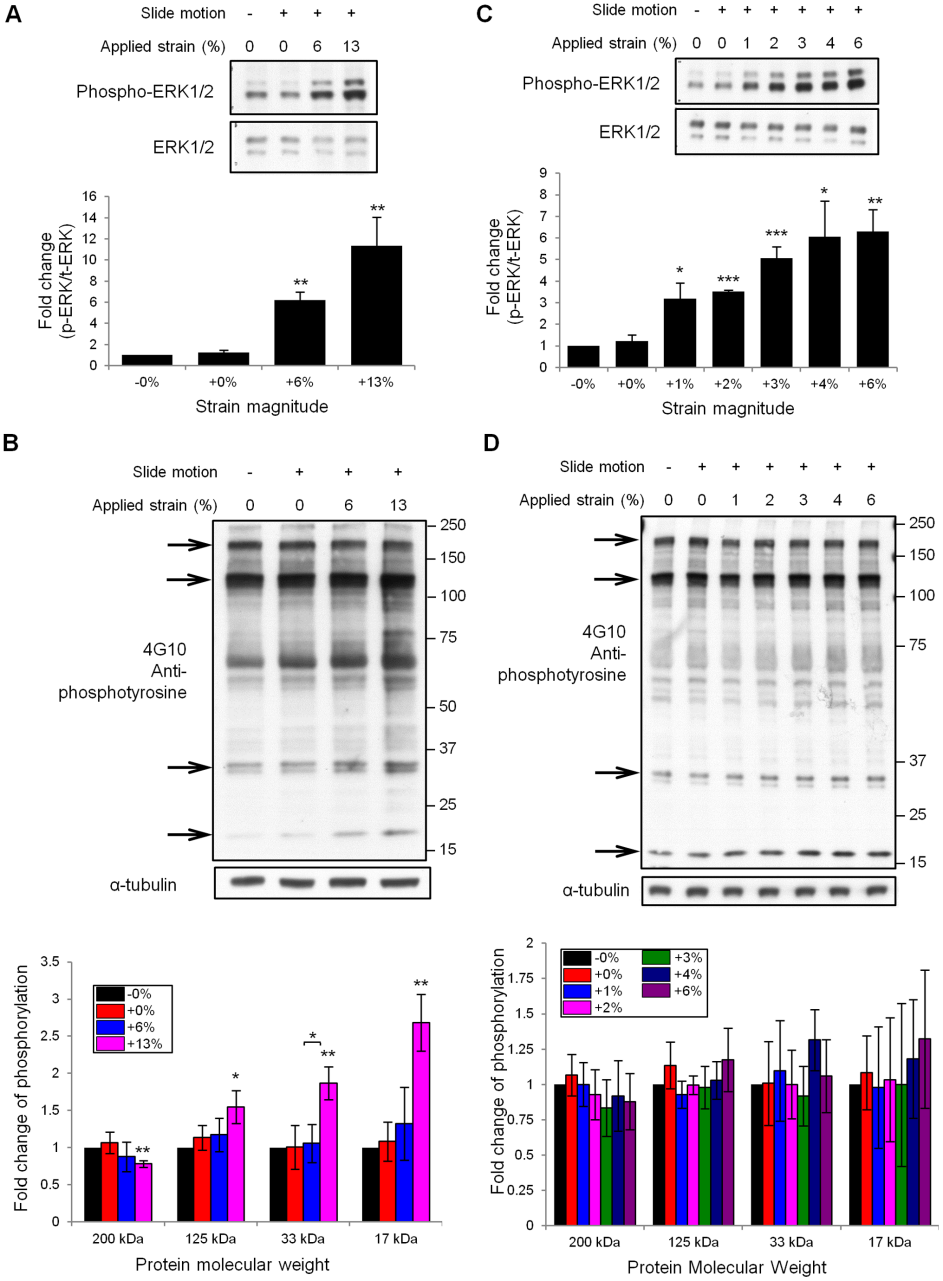
Immunoblot analysis of cell signaling induced by cyclic stretching. (A) RPTP-α^+/+^ fibroblasts cultured on collagen-coated membranes were subjected to no mechanical stimulus (lane 1), periodic vertical motion with no strain (lane 2), and cyclic strains of 6% (lane 3) and 13% (lane 4), at a frequency of 0.5 Hz for 30 minutes. While no change was observed in the levels of ERK expression, its phosphorylation level increased in lanes 3 and 4 as compared to the first two. At least three independent experiments were conducted to quantify stretch-dependent ERK phosphorylation. Paired Student’s *t*-tests showed that cell stretching produced a significant increase in ERK phosphorylation (**p<0.01, *n* = 3). (B) Anti-phospho-tyrosine immunoblot analysis showed different strain responses among individual tyrosine-phosphorylated proteins. Arrows indicate bands at molecular weights of 200 kDa, 125 kDa, 33 kDa and 17 kDa, respectively. The signal intensity from these bands was normalized against the corresponding α-tubulin intensity, and the mean values of the fold change in tyrosine phosphorylation from three independent experiments were plotted. At 13% strain, the 200 kDa band showed a significantly decreased intensity, while the intensity of the other three bands (125 kDa, 33 kDa and 17 kDa) increased significantly as compared to the unstrained control (* <0.05, **p<0.01, paired Student’s *t*-tests, *n* = 3). (C) MEFs subjected to cyclic strain ranging from 1% to 6% (0.5 Hz, 30 min) exhibited a stepwise increase in the level of ERK phosphorylation in response to strain. All the stretched samples showed a statistically significant increase in ERK phosphorylation relative to the unstrained control (*p<0.05, **p<0.01, ***p<0.005, paired Student’s *t*-tests, *n* = 3). (D) At small magnitudes (≤6%) of cyclic strain, tyrosine phosphorylation did not show significant changes from the unstrained control. Note: All error bars in this figure indicate standard deviations.

**Figure 7:**
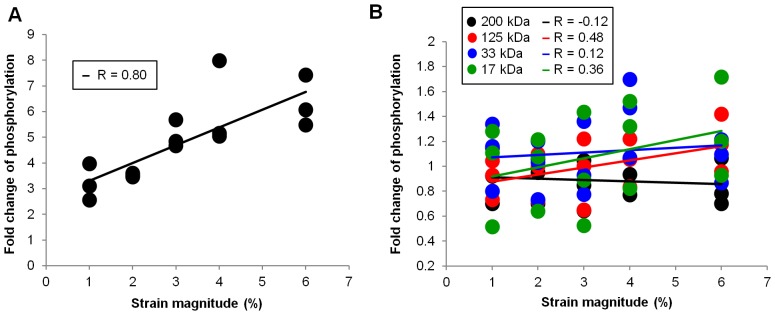
Correlation between strain magnitudes and phosphorylation level. (A) Scatterplot of ERK phosphorylation levels against applied strains, obtained from three independent experiments (Figure 6C). Pearson’s correlation coefficient (R) was calculated and found to be statistically significant (R = 0.80, *n* = 15, *p*<0.01). (B) Scatterplot of tyrosine phosphorylation levels against applied strains, obtained from three independent experiments (Figure 6D). Pearson’s correlation coefficients between phosphorylation level and strain magnitude were calculated for four bands (200 kDa, 125 kDA, 33 kDa, 17 kDa) in the phosphotyrosine blot (Figure 6D). The 125 kDa and 17 kDa bands showed moderate positive correlation with applied strain magnitude (R = 0.48 and R = 0.36, respectively). However, none of the coefficients were statistically significant (*n* = 15, *p*>0.05 for all R).

Samples were loaded onto NuPAGE Novex 4–12% Bis-Tris gels (Invitrogen, Carlsbad, CA) for separation by gel electrophoresis. Separated proteins were transferred to nitrocellulose membranes, which were then incubated with a blocking buffer for 1 hour at room temperature and probed with specific primary antibodies. After washing 3 times with TBS-T (20 mM Tris pH 7.4, 137 mM NaCl, 0.1% Tween-20), the membranes were incubated with the appropriate secondary antibodies conjugated with horseradish peroxidase, and washed 5 times with TBS-T. An enhanced chemi-luminescence (ECL) system (Thermo Scientific Pierce Protein Biology, Rockford, IL) was used to visualize the antibody binding. The rabbit polyclonal anti-ERK1/2 and anti-phospho-ERK1/2 antibodies were purchased from Cell Signaling Technology (Danvers, MA), and the mouse monoclonal anti-phospho-tyrosine antibody (4G10) was purchased from Merck Millipore (Billerica, MA). The blots were quantified using NIH ImageJ software. Various strain magnitudes were compared to the unstrained controls using paired Student’s *t*-tests. To analyze the incremental effects of low strains (1%–6%) on ERK and tyrosine phosphorylation (Figures 6C and 6D), we calculated the Pearson’s correlation coefficient [23] between these two variables (Figure 7).

## Results

### A 1 mm Thick Wall Gives Better Strain Uniformity at the Wall-membrane Junction than a 0.5 mm Thick Wall

To design a stretchable culture well causing minimal strain heterogeneity, we conducted finite element simulations in which we varied the wall thickness and the wall height ((i) and (ii) in Figure 2A) and calculated the resultant strain profiles. The values for wall thickness and height that produced less heterogeneous strain profiles were determined as the dimensions of the PDMS chambers, and the mold was designed accordingly.

The simulation results obtained by varying the thickness of the wall are presented in Figure 2B –2C. The normalized radial strain (*e_r_*/*e_r0_*) profiles for both values of thickness (Design 1∶0.5 mm; Design 2∶1 mm) near the wall interface were computed. The radial strains were normalized with respect to *e_r0_*, the radial strain at the center of the membrane (*r = *0) to facilitate comparison between Designs 1 and 2. As shown in Figure 2C, Design 2 produced a more uniform radial strain profile near the membrane-wall junction (indicated by red arrows in Figure 2A). These observations led us to select the wall thickness to be 1 mm. Although a further increase in wall thickness might improve strain uniformity, walls thicker than 1 mm were not considered for fear of a possible escalation of forces required to produce strains.

Furthermore, simulation results showed that varying the wall height did not have a significant effect upon the strain uniformity (unpublished data). As such, the wall height was defined to be 10 mm.

### Uniform Biaxial Strains are Generated upon Membrane Stretching

Membranes were stretched over the indenter by displacing the holding plate (Figure 3D) downward stepwise, and returned to their original position along the same path, creating a loading-unloading cycle. At each step in the cycle, the radial (E_rr_), circumferential (E_cc_) and shear (E_rc_) components of strain on the membrane were calculated (see Methods). Plots of the mean and standard deviation of E_rr_, E_cc_ and E_rc_ against the corresponding displacement of the holding plate in a single loading-unloading cycle are shown in Figure 4B–4D.

At each displacement step, the strain components on the four membranes had similar magnitudes (Figure 4B–4D), indicating consistency in the material properties across all the membranes tested. For each membrane, the radial and circumferential strains values at a given displacement of the holding plate were almost equal. This was observed along both the loading and the unloading cycle (Figure 4B, 4C). In line with this equivalence of radial and circumferential strains, the mean shear strain values were consistently close to zero regardless of the holding plate displacement (Figure 4D).

Radial and circumferential strains exhibited a small degree of hysteresis in the loading-unloading cycle (Figure 4B, 4C). Magnitudes of these strains along the unloading cycle (see blue arrows in Figure 4B–4D) were slightly larger than those along the loading cycle (red arrows in Figure 4B–4D), for the same amount of membrane displacement. This difference was more prominent at larger displacements. As the displacement decreased, the trajectories of the loading and unloading cycles appeared to become closer to each other. When the membrane was brought back to the zero position, the strain returned to zero. This indicates that when the membrane is subjected to cyclic strain, the endpoints, i.e. maximum and zero strains, are consistently reproduced despite slight differences in the paths taken between loading and unloading cycles.

To further the analysis of the strain distribution uniformity, we plotted color intensity maps of radial and circumferential strains over the surface of the membrane, at holding plate displacements of 3.5 mm, 5.5 mm, and 9.5 mm, during the loading phase. As shown in the columns labeled E_rr_ and E_cc_ in Figure 4E, there were small differences between the radial and circumferential strain values from point-to-point across the area on which cells are cultured. To normalize these differences, we computed the coefficient of variation (CV, defined as the ratio of the standard deviation to the mean) over the area of cell culture. The CV values range from 0.06 to 0.09, and are thus on the order of the CV values in the central region of existing equibiaxial stretching devices (Table S1).

### Cells Adhere to the Custom-molded PDMS, and can be Imaged on the Stretching Device

MEFs and HEK293 cells grown on the collagen-coated PDMS membranes for 24 h are shown in Figure 5A (top). They adhered to the membranes and spread on them in a similar manner to when grown on standard culture plastics (polystyrene) (Figure 5A, bottom).

In addition, we were able to observe cell morphology before and after stretching, using an inverted microscope. As shown in Figure 5B, the cell dimension increased upon application of substrate stretching, indicating that strains were transmitted from the substrate to the cells spread on it.

### Cyclic Stretching Modifies ERK and Tyrosine Phosphorylation in a Strain Magnitude-dependent Manner

To validate the biochemical analysis of stretch response of cells using our device, we examined ERK and tyrosine phosphorylation, both of which have been reported to be stretch-responsive [17]–[19]. For this study, two controls were used: one kept static (Figure S1A) and the other cycled vertically in the absence of a strain-inducing indenter (Figure S1B).

As shown in Figure 6A, ERK was phosphorylated by cyclic cell stretching (0.5 Hz, 30 min) depending on the strain magnitude, whereas the total ERK levels were not changed. Notably, there was no significant difference observed in the ERK phosphorylation between the two control samples (compare lanes 1 and 2 in Figure 6A). This suggests that the ERK phosphorylation, observed after 6% and 13% stretching (lanes 3 and 4 in Figure 6A), specifically resulted from stretching rather than from other causes such as membrane vibration. The change in ERK phosphorylation caused by cell stretching was quantified and found to be statistically significant relative to the unstrained control (Figure 6A).

While tyrosine phosphorylation levels also showed strain-dependent changes, their responses appeared to differ among individual tyrosine-phosphorylated proteins (refer to bands at 200, 125, 33 and 17 kDa indicated by arrows in Figure 6B). We analyzed these differences by quantifying individual band intensities with reference to the unstrained control. At 13% strain, the band intensity at 200 kDa was significantly lower compared to the unstrained control (Figure 6B). In contrast, the band intensities at 125 kDa, 33 kDa, and 17 kDa showed significant increases at 13% strain (Figure 6B). These results suggest that different mechano-responsive molecules have different strain sensitivity.

One of the key advantages of this device lies in the ability to test the effects of very small magnitudes of strain with minimal strain inhomogeneity. We therefore applied small magnitudes of strains ranging from 1% to 6% using our device (0.5 Hz, 30 min). We found that ERK phosphorylation was increased even by a strain as low as 1% (lane 3 in Figure 6C). Furthermore, we observed stepwise increases in ERK phosphorylation from 1% strain to 6% strain (lanes 3–7 in Figure 6C), although the Student’s t-test did not show statistical significance for the increase in relative phosphorylation level from one strain magnitude to another (for example, from 1% to 2%, from 3% to 4% or from 4% to 6%). Still, when we analyzed the incremental effects of this range of strains on ERK and tyrosine phosphorylation by computing Pearson’s correlation coefficient (R) [23], there was a strong positive correlation between strain magnitude and relative ERK phosphorylation (R = 0.80, *n* = 15, *p*<0.01, Figure 7A). The significant consistency (*p*<0.01) between the increase in ERK phosphorylation levels and incremental strain magnitudes (Figure 7A) suggests that the cells were sensitive to the differences in strain magnitudes within the range exerted by our cell stretching system. In contrast, tyrosine phosphorylation did not show any significant changes in response to strains up to 6% (Figure 6D). The Pearson’s correlation coefficients between incremental strain magnitudes and relative tyrosine phosphorylation levels were not statistically significant (Figure 7B). In addition, similar results were obtained using a ranking analysis of the strain responses (Table S2).

## Discussion

### Inhomogeneous Substrate Strains in Existing Culture Devices

The primary objective of this study was to improve upon the existing limitations in equibiaxial cell stretching devices (Figure 1A, 1B). To address these limitations, Xie *et al.* [15] used finite element modeling to analyze the strain field on a polyurethane membrane deformed by an indenter. They found that while strain remained homogeneous in the region of the membrane within the indenter, the peripheral region beyond the indenter exhibited a gradual increase in radial strain and decrease in circumferential strain as distance from the center increased. The authors concluded that the strain field is equibiaxial in the center but increasingly uniaxial towards the periphery. Therefore, cells are subjected to a mixture of equibiaxial and uniaxial strains when stretched using such devices.

The biochemical response of cells has been reported to differ between uniaxial and equibiaxial stretching. Kaunas *et al.* [24] have shown that c-Jun N-terminal kinase (JNK) is only transiently activated upon cyclic uniaxial stretching, but that activation is sustained under biaxial stimuli. Lee *et al.* [25] demonstrated that in adult rat cardiac fibroblasts, uniaxial strain produced increased activation of transforming growth factor (TGF)-β1 as compared to biaxial strain, whereas the inverse was true when the strain magnitudes were increased.

It is clear from the above studies [24], [25] that the cellular responses to uniaxial and biaxial stretching differ. Moreover, as Xie *et al.* [15] have shown, the design used in many existing equibiaxial stretching devices produces a mixture of equibiaxial and uniaxial strains. Therefore, when biochemical analysis is conducted using cell samples stretched upon such systems, the readout is likely to be the average of cell responses to two differing stimuli, indicating a need to improve the current design to eliminate strain heterogeneity.

### Problems in Current Methods to Address Strain Homogeneity

Existing studies have used either design or methodological workarounds to address the issue of heterogeneous substrate strains. In one study [12], the membrane was cut into two pieces to separate the central region of the membrane from the outer region prior to cell lysis. However, cutting the membrane introduces undesirable mechanical forces on the cells. These perturbations might reduce experimental sensitivity, particularly when the objective is to study the effects of one-time stretching.

Some studies have instead confined cells and culture medium to the central region of the membrane by using separately placed rings or inserts [14], [26]. These rings are removed prior to strain application and culture medium added if required. Nevertheless, we believe that incorporating a wall makes sample collection after strain application easier, as it defines a physical boundary for washing and lysing the cells. Having a wall also eliminates any need to add culture medium during strain application. In addition, we expect external culture rings to be more prone to medium leakage, a problem that we could completely eliminate by physically incorporating the wall into the membrane.

In other designs, the indenter was placed on top of the cell layer and the membrane stretched upward [25], [27], [28]. In this setup, greasing of the indenter would not be advisable as the grease may contaminate culture medium and influence cell behavior. Consequently, the lack of grease would increase frictional contact between membrane and indenter. Moreover, cells at the boundary would be sheared between the indenter and the membrane during stretching (Figure S2).

Waters *et al*. [16] designed a system which implemented biaxial, in-plane stretching upon a cruciform membrane. Separately cast rings of PDMS were affixed to the central region of the membrane. This allowed cells to be cultured within a region that sustained homogeneous strains, similar to our design. However, our device improves upon two major limitations in this system. First, to create culture wells on the membrane surface, Waters *et al.* placed fully cured, thin-walled (∼0.1 mm thick) PDMS rings onto the surface of a partially cured membrane. They presumed that complete curing of the membrane reliably fuses the rings onto the membrane surface. However, the joint between membrane and ring is likely to sustain greater stresses due to the change in geometry at this location. The ring may therefore be subject to detachment from the membrane, which might lead to leakage of culture medium, thereby making it impossible to retain cell culture. Furthermore, the strain profile within the well may change upon ring detachment. This would be a concern particularly when the structure is subjected to a high degree of strain over an extended period of time. In our design, the well and the membrane are molded together as a continuous structure, providing a stronger joint than merely fusing the two parts together. Indeed, we have not experienced well detachment even after applying cyclical strains of magnitude 13% (Figure 6A).

Second, in Waters’ system, the membrane is suspended between four clamps on the side plates. This might cause sagging of the membrane in the center. In contrast, our clamping design applies a slight pre-stress to the clamped membrane to prevent sagging. This reduces cell accumulation in the center and also ensures that applied strains are more reliable, particularly at small magnitudes (e.g. 1% to 6%).

While our device was designed specifically to overcome limitations associated with biochemical analysis, it does not permit us to perform high-resolution microscopy of stretched samples. In particular, our mold produces a membrane of thickness 0.5 mm, which precludes imaging using objectives of high numerical aperture, whose working distances are typically on the order of 0.13 mm to 0.17 mm.

### Validation of Strain Homogeneity and Uniformity of Membrane Material Properties

We examined the measured strain distribution over the membrane surface by plotting color intensity maps of strain at different magnitudes of stretching. Radial strains appeared to increase adjacent to the wall-membrane junction (Figure 4E), consistent with the predictions from our finite element simulations (blue line in Figure 2C). However, when examined across the entire culture surface, the distribution of both radial and circumferential strains appeared homogeneous. The coefficient of variation (CV) values, which represent the extent of strain variation, are similar to those in existing equibiaxial stretching devices (Table S1). This indicates that the variation in strain over the culture region in our device is similar to that in the uniformly stretched central region of existing equibiaxial stretching devices. In all, the fraction of cells subjected to heterogeneous strains in our device is expected to be smaller compared to that in an existing alternative (Supporting Information S1).

Unlike other systems which use commercially manufactured membranes [9]–[12], we cast our membranes in-house. Individual casting of each membrane raises the possibility of differences in their material properties. However, strain analysis of the membrane surface showed that this was not the case. Data for the radial, circumferential, and shear strains on four different membranes plotted in Figure 4B –4D showed overlap, indicating consistency in the material properties of the membrane.

### Strain-dependent Changes in Protein Phosphorylation Levels

To validate our device, we first subjected cells to strains expected to induce mechano-responsive intracellular signal transduction. Applying cyclic strains of 6% and 13% (0.5 Hz, 30 min) resulted in a strain-dependent increase in phosphorylation of ERK (Figure 6A). ERK phosphorylation drives the family of AP-1 transcription factors for a pro-growth response, and has been reported to be up-regulated by integrins upon stretching [17]. It was therefore tested to confirm appropriate functioning of our device. Sotoudeh *et al.* [12] have reported a strain magnitude-dependent increase in AP-1/TRE mediated transcriptional activation, which lies downstream of ERK phosphorylation, upon cyclic stretching. Therefore our findings show consistency with existing reports.

We next examined tyrosine phosphorylation, which is implicated in the activation of a number of focal adhesion proteins (e.g. focal adhesion kinase, paxillin, p130Cas) [19]. The changes in tyrosine phosphorylation levels upon application of strain appeared to vary among individual tyrosine-phosphorylated proteins. At 13% strain, three of the bands analyzed (125 kDa, 33 kDa and 17 kDa) showed an increase in intensity whereas one (200 kDa) showed decreased intensity as compared to the unstrained control (Figure 6B). This indicates that different mechano-sensitive molecules have varying strain sensitivity, in line with a previous report describing different responses of NF-κB to different magnitudes of stretching [29].

One of the key advantages of our device is the ability to uniformly apply small magnitudes of equibiaxial strain. Previous studies have investigated the effects of small magnitudes of uniaxial strain. Jungbauer *et al*. [30] have reported fibroblast reorientation on substrates subjected to cyclic uniaxial stretching, at strain magnitudes exceeding 1%. Recently, Leong *et al*. [31] reported that cyclic tensile loading at small magnitudes of strain can regulate differentiation of human mesenchymal stem cells (hMSCs). In agreement with these studies, we observed an increase in ERK phosphorylation in RPTP-α^+/+^ MEFs at strains as small as 1%, which consistently increased stepwise up to a strain of 6% (Figure 6C, Figure 7A). However, these magnitudes of strain did not induce a significant change in tyrosine phosphorylation (Figure 6D, Figure 7B). This clearly suggests that in RPTP-α^+/+^ MEFs ERK is more sensitive than tyrosine phosphorylation to the magnitude of externally applied mechanical strain.

From other studies, as well as our own, it seems evident that cells are sensitive to small magnitudes of strain (Figure 6C, Figure 7A). However, these effects appear to have been investigated mainly in the context of uniaxial strains [30], [31]. As discussed before, when substrate strains are uniaxial, cells which are randomly oriented are inevitably subjected to heterogeneous and anisotropic strains. To exclude anisotropy and heterogeneity, and specifically study the effects of strain magnitude, equibiaxial strains are preferred. Our device provides a means to accurately and uniformly impart small magnitudes of equibiaxial strains to cells and perform biochemical analysis of the consequent cellular responses. Using our device, we have been able to elucidate differences in strain sensitivity between ERK and tyrosine phosphorylation, both of which are reportedly regulated by mechanical strain [17]–[19].

## Supporting Information

Figure S1 **Controls used in cyclic stretching experiments.**
(PDF)

Figure S2 **Inverting the configuration of indenter and membrane leads to cell shearing at the boundary.**
(PDF)

Figure S3 **Manual device for application of static equibiaxial strains.**
(PDF)

Table S1 **Coefficients of variation for strain distribution in previously described equibiaxial stretching devices.**
(PDF)

Table S2 **Ranking analysis of cellular response to small strain magnitudes using the Page test for ordered alternatives.**
(PDF)

Supporting Information S1 **Strain heterogeneity in existing equibiaxial stretching devices.**
(PDF)
