# Supplementary material for: Design and Construction of an Equibiaxial Cell Stretching System That Is Improved for Biochemical Analysis
Source: PLoS One. 2014 Mar 13;9(3):e90665. doi: 10.1371/journal.pone.0090665 (PMC3953117; doi:10.1371/journal.pone.0090665)
Supplement: Figure S1 — Controls used in cyclic stretching experiments. (PDF) [file pone.0090665.s001.pdf]

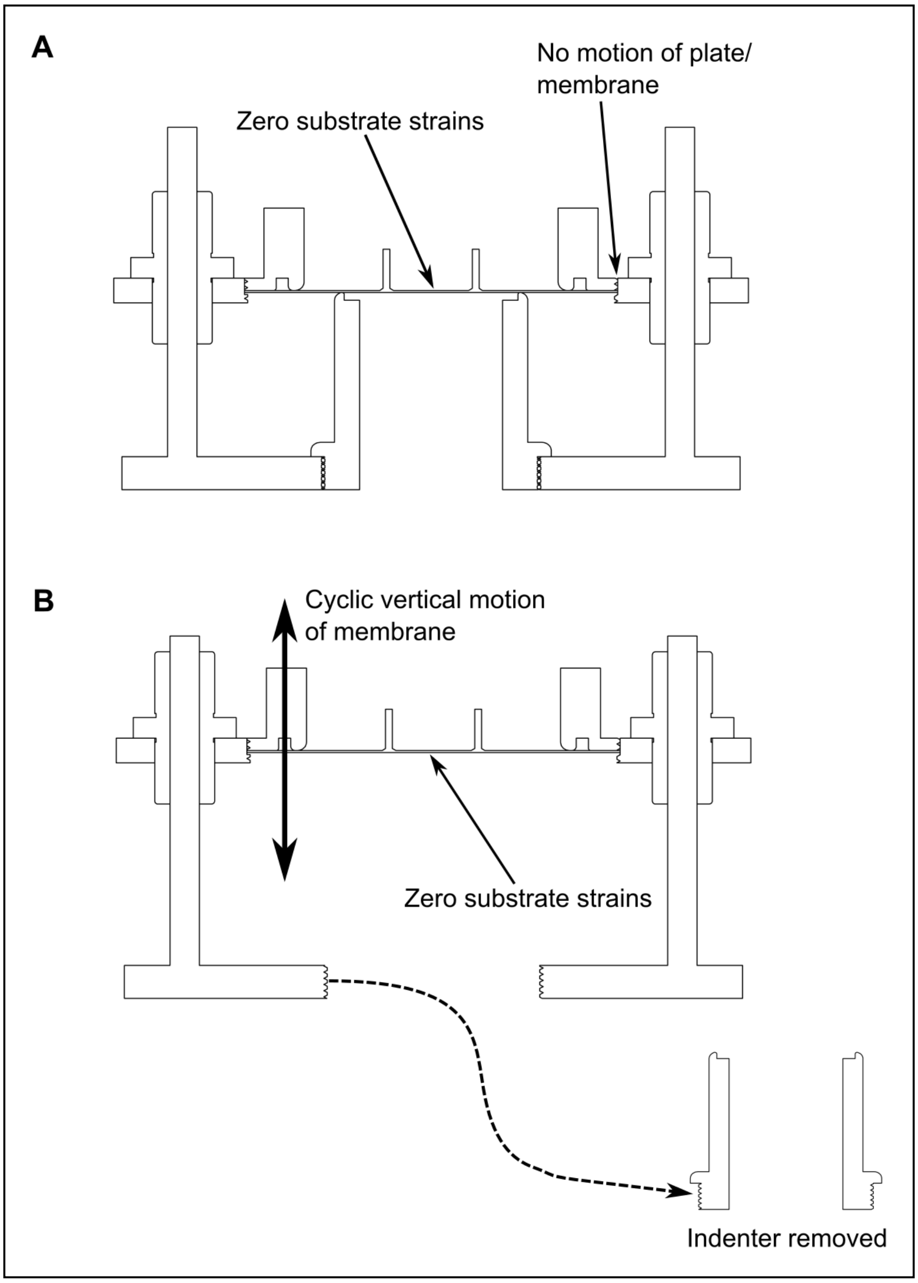

Figure S1. Controls used in cyclic stretching experiments. (A) In the 'static' control, the membrane holder was engaged with the stretching device and allowed to stand for the appropriate period of time. The membrane was not stretched and no displacement of the holding plate took place. (B) In the 'dynamic' control, the indenter was removed from the device. Then the membrane holder was engaged and the holding plate was cycled vertically for 30 minutes at 0.5 Hz. The vertical displacement was equivalent with that used for 6% stretching.
