# Supplementary material for: Design and Construction of an Equibiaxial Cell Stretching System That Is Improved for Biochemical Analysis
Source: PLoS One. 2014 Mar 13;9(3):e90665. doi: 10.1371/journal.pone.0090665 (PMC3953117; doi:10.1371/journal.pone.0090665)
Supplement: Figure S2 — Inverting the configuration of indenter and membrane leads to cell shearing at the boundary. (PDF) [file pone.0090665.s002.pdf]

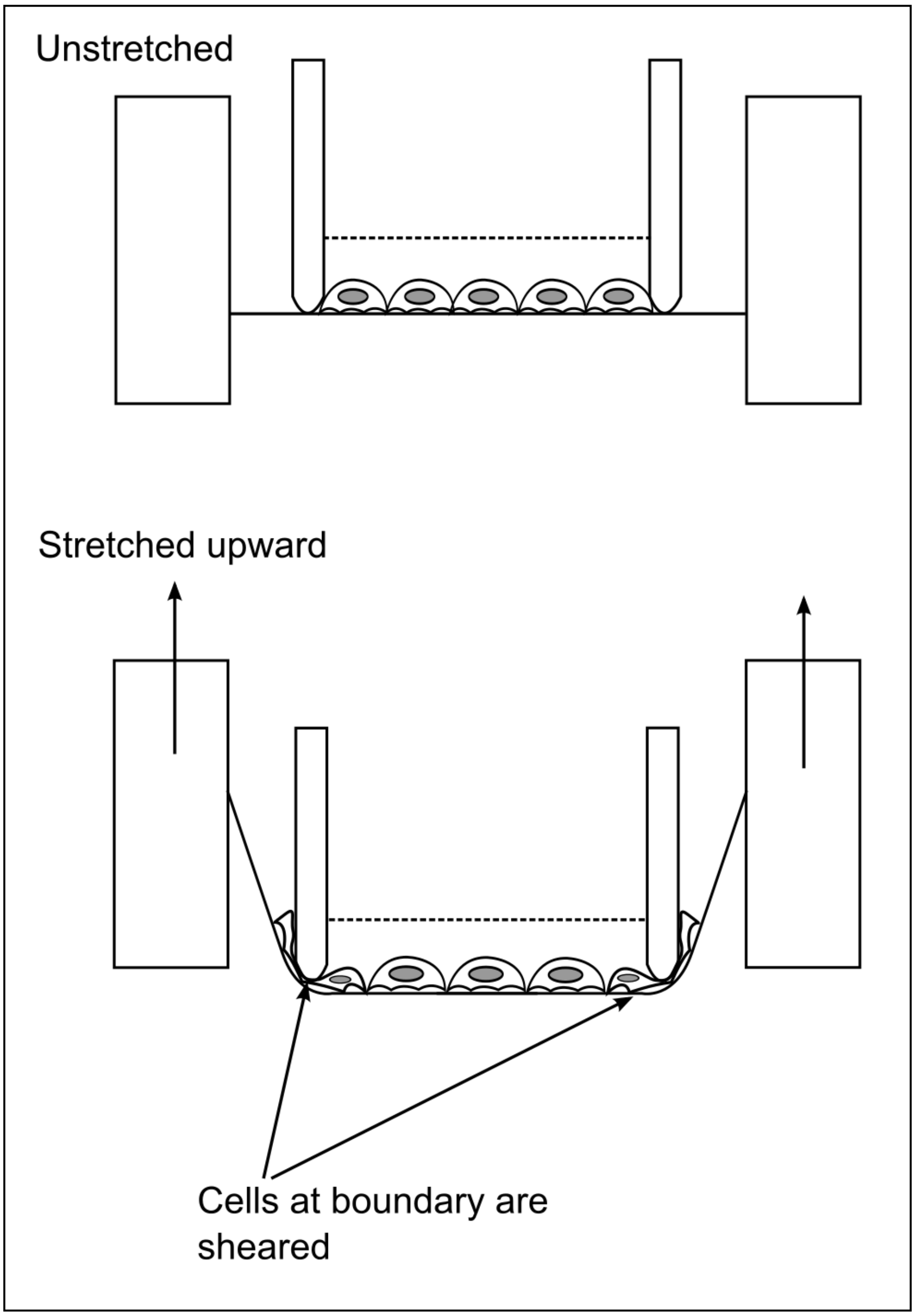

Figure S2. Inverting the configuration of indenter and membrane leads to cell shearing at the boundary. In some of the existing devices, the indenter is placed on the upper surface of the membrane. While a well for cell culture is created by the indenter, cells on the periphery may undergo shearing between the indenter and the membrane upon stretching.
