# Supplementary material for: Design and Construction of an Equibiaxial Cell Stretching System That Is Improved for Biochemical Analysis
Source: PLoS One. 2014 Mar 13;9(3):e90665. doi: 10.1371/journal.pone.0090665 (PMC3953117; doi:10.1371/journal.pone.0090665)
Supplement: Figure S3 — Manual device for application of static equibiaxial strains. (PDF) [file pone.0090665.s003.pdf]

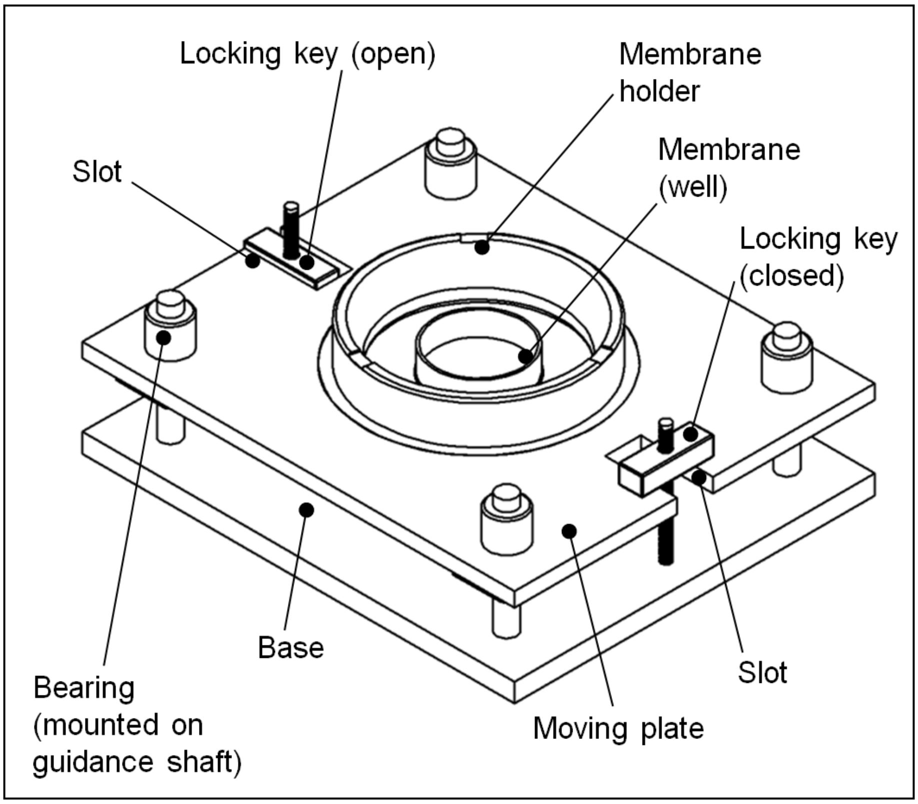

Figure S3. Manual device for application of static equibiaxial strains. A separate device was designed and constructed to permit manual strain application to the membranes, without requiring a motor. In this device, an 8 mm thick, 18 cm × 15 cm plate served as a base, and possessed a replaceable indenter in the center and a guidance shaft at each corner. A 6 mm thick holding plate moved along the pins by means of ball bearings, similar to the motor-operated device. The center of the plate was cut and threaded to engage membrane holders. Both membrane holders and indenters were interchangeable between the manually operated device and the motorized device.

Upon stretching, the restorative tension in the membrane needs to be counterbalanced to maintain strain. For this purpose, the manual device featured a locking and positioning system. Slots were cut into the holding plate at the midpoints of the shorter edges. Screws were threaded at the corresponding position on the base, protruding through the center of the slot. Aluminum keys possessing a female thread in the center were engaged with these screws. To apply a fixed membrane strain, the keys were moved along the screws to a fixed distance from the base. Initially, they were positioned parallel to the slots, allowing the holding plate to move past them. Once the plate was moved past, the keys could be rotated at right angles to lock the plate in position.
