# Supplementary material for: Design and Construction of an Equibiaxial Cell Stretching System That Is Improved for Biochemical Analysis
Source: PLoS One. 2014 Mar 13;9(3):e90665. doi: 10.1371/journal.pone.0090665 (PMC3953117; doi:10.1371/journal.pone.0090665)
Supplement: Table S1 — Coefficients of variation for strain distribution in previously described equibiaxial stretching devices. (PDF) [file pone.0090665.s004.pdf]

Table S1: Coefficients of variation for strain distribution in previously described equibiaxial stretching devices

Source	Radial strain			Circumferential strain		
	Mean	SD	CV	Mean	SD	CV
Schaffer et al. *J Orthop Res* 12(5): 709-719, 1994 [1]	0.093	0.009	**0.10**	0.091	0.006	**0.07**
Lee et al. *Am J Physiol* 271: C1400-C1408, 1996 [2]	0.035	0.003	**0.09**	0.032	0.003	**0.09**
	0.108	0.006	**0.06**	0.104	0.007	**0.07**
Sotoudeh et al. *Ann Biomed Eng* 26: 181-189, 1998 [3]	0.037	0.003	**0.08**	0.036	0.002	**0.06**
	0.129	0.009	**0.07**	0.127	0.008	**0.06**

Values for mean and standard deviation (SD) of radial and circumferential strain components were obtained from published work [1-3] characterizing equibiaxial stretching devices. The coefficient of variation (CV) for the strain distribution in these devices was then calculated as the ratio of SD to mean. The CV values represent the variation in strain within the homogeneously stretched central region of these stretching devices and range from 0.06 to 0.10. The CV values calculated for our device (Figure 4E) fall within this range, suggesting that variation in strain within the culture region in our device is similar to that in the central region of existing equibiaxial stretching devices. However, in our device, all cells are stretched homogeneously, while in existing devices a fraction of cells lies in the peripheral region, which is stretched heterogeneously (Supporting Information S1).

REFERENCE

1. Schaffer JL, Rizen M, L'Italien GJ, Benbrahim A, Megerman J, et al. (1994) Device for the application of a dynamic biaxially uniform and isotropic strain to a flexible cell culture membrane. J Orthop Res 12: 709–719.
2. Lee AA, Delhaas T, Waldman LK, MacKenna DA, Villarreal F, et al. (1996) An equibiaxial strain system for cultured cells. Am J Physiol Cell Physiol 271: C1400–C1408.
3. Sotoudeh M, Jalali S, Usami S, Shyy JY, Chien S (1998) A strain device imposing dynamic and uniform equi-biaxial strain to cultured cells. Ann Biomed Eng 26: 181–189.
