# Supplementary material for: Design and Construction of an Equibiaxial Cell Stretching System That Is Improved for Biochemical Analysis
Source: PLoS One. 2014 Mar 13;9(3):e90665. doi: 10.1371/journal.pone.0090665 (PMC3953117; doi:10.1371/journal.pone.0090665)
Supplement: Table S2 — Ranking analysis of cellular response to small strain magnitudes using the Page test for ordered alternatives. (PDF) [file pone.0090665.s005.pdf]

Table S2: Ranking analysis of cellular response to small strain magnitudes using the Page test for ordered alternatives

		H1		H2	
**Protein**	***n***	**Test statistic *L***	***p*-value**	**Test statistic *L***	***p*-value**
ERK phosphorylation	3	417	< 0.001	255	> 0.05
Tyrosine phosphorylation 200 kDa band	3	331	> 0.05	341	> 0.05
Tyrosine phosphorylation 125 kDa band	3	345	> 0.05	327	> 0.05
Tyrosine phosphorylation 33 kDa band	3	357	> 0.05	315	> 0.05
Tyrosine phosphorylation 17 kDa band	3	366	> 0.05	306	> 0.05

To examine whether ERK and tyrosine phosphorylation responded to a stepwise small increase in strain magnitude, the band intensities for all the strains (0% – 6%) in each blot for Figure 6C and 6D were ranked and analyzed by the Page test for ordered alternatives ($n = 3$) [23]. The Page test was used to assess the null hypothesis that there was no difference in the band intensities, against the predicted alternatives that the band intensities showed an ordered change (i.e., increase or decrease) with incremental strain. Thus,

Null hypothesis H0: $P_{0-} = P_{0+} = P_1 = P_2 = P_3 = P_4 = P_6$,
Alternative hypothesis H1: $P_{0-} \leq P_{0+} < P_1 < P_2 < P_3 < P_4 < P_6$,
Alternative hypothesis H2: $P_{0-} \geq P_{0+} > P_1 > P_2 > P_3 > P_4 > P_6$,

Here P indicates the rank of the band intensity and the subscript indicates the strain condition. We calculated the Page statistic for hypotheses H1 and H2 against the null hypothesis H0, for both ERK and tyrosine phosphorylation. The results showed that in the case of ERK phosphorylation, hypothesis H1 would hold against H0 with a high degree of confidence ($p < 0.001$), indicating a consistent increase in ERK phosphorylation with strain. In contrast, for the 200 kDa, 125 kDa, 33 kDa and 17 kDa bands analyzed in Figure 6D, neither hypothesis H1 nor H2 held against H0, suggesting that stepwise increase in strain up to 6% produced neither a consistently significant increase nor decrease in phosphorylation levels.
