# Supplementary material for: Design and Construction of an Equibiaxial Cell Stretching System That Is Improved for Biochemical Analysis
Source: PLoS One. 2014 Mar 13;9(3):e90665. doi: 10.1371/journal.pone.0090665 (PMC3953117; doi:10.1371/journal.pone.0090665)
Supplement: Supporting Information S1 — Strain heterogeneity in existing equibiaxial stretching devices. (PDF) [file pone.0090665.s006.pdf]

Supporting Information S1: Strain heterogeneity in existing equibiaxial stretching devices

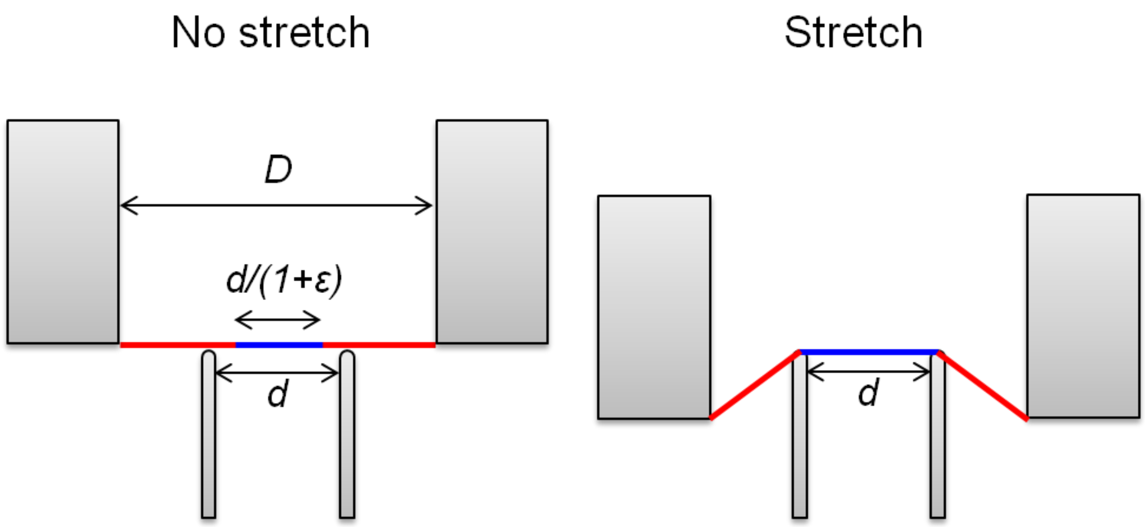

Figure S1.1: Factors affecting fraction of uniformly stretched cells in existing devices. In existing devices, cells are cultured over the entire surface of the device, which has a diameter *D*. Cells are stretched using an indenter of diameter *d*, to a strain ε. Only cells in the central region of the membrane (shown in blue) are subjected to homogeneous strains. This region has a diameter $d/(1+\varepsilon)$ in the unstrained condition (left), and expands to a diameter *d* when stretched (right). Cells attached to the remaining membrane area (in red) experience heterogeneous strains.

Discussion

Heterogeneous strains in the peripheral region of the membrane cannot be experimentally quantified due to the curved shape of this region upon stretching. Instead, the fraction of cells cultured in this region is used to yield a quantifiable measure of heterogeneity. Using the dimensions illustrated in Figure S1.1, this fraction can be described by the following equation:

$$1 - d^2/D^2(1+\varepsilon)^2 \qquad (1)$$

Equation (1) was used to examine strain heterogeneity in the commercially available Flexcell FX-5000 device (Table S1.1), based on its reported dimensions [4]. However, as dimensions for other devices were unavailable, they could not be examined. Instead, we used known dimensions of our own device, while assuming that it was fitted with a flat membrane instead of a membrane with a central well. The results of the calculations are tabulated below.

Table S1.1: Fraction of cells subjected to heterogeneous strain (ε = 0.1) in devices using existing designs.

Stretching device	***D*** **(mm)**	***d*** **(mm)**	Fraction of cells subjected to heterogeneous strains
Flexcell FX-5000	35 [1]	25 [1]	0.58
Our device with flat membrane	68	45	0.64
Our device with flat membrane and wide indenter	68	60	0.36

REFERENCE

1. Flexcell International Corporation (2009) Flexcell FX-5000 Tension System. Available: http://www.flexcellint.com/documents/FX-5000TensionUsersManual.pdf.
